# Supplementary material for: Non-invasive optoacoustic imaging of dermal microcirculatory revascularization in diet-induced obese mice undergoing exercise intervention
Source: Photoacoustics. 2024 Jun 30;38:100628. doi: 10.1016/j.pacs.2024.100628 (PMC11269314; doi:10.1016/j.pacs.2024.100628)
Supplement: Supplementary file 1 — Supplementary material [file mmc1.docx]

Supplementary files


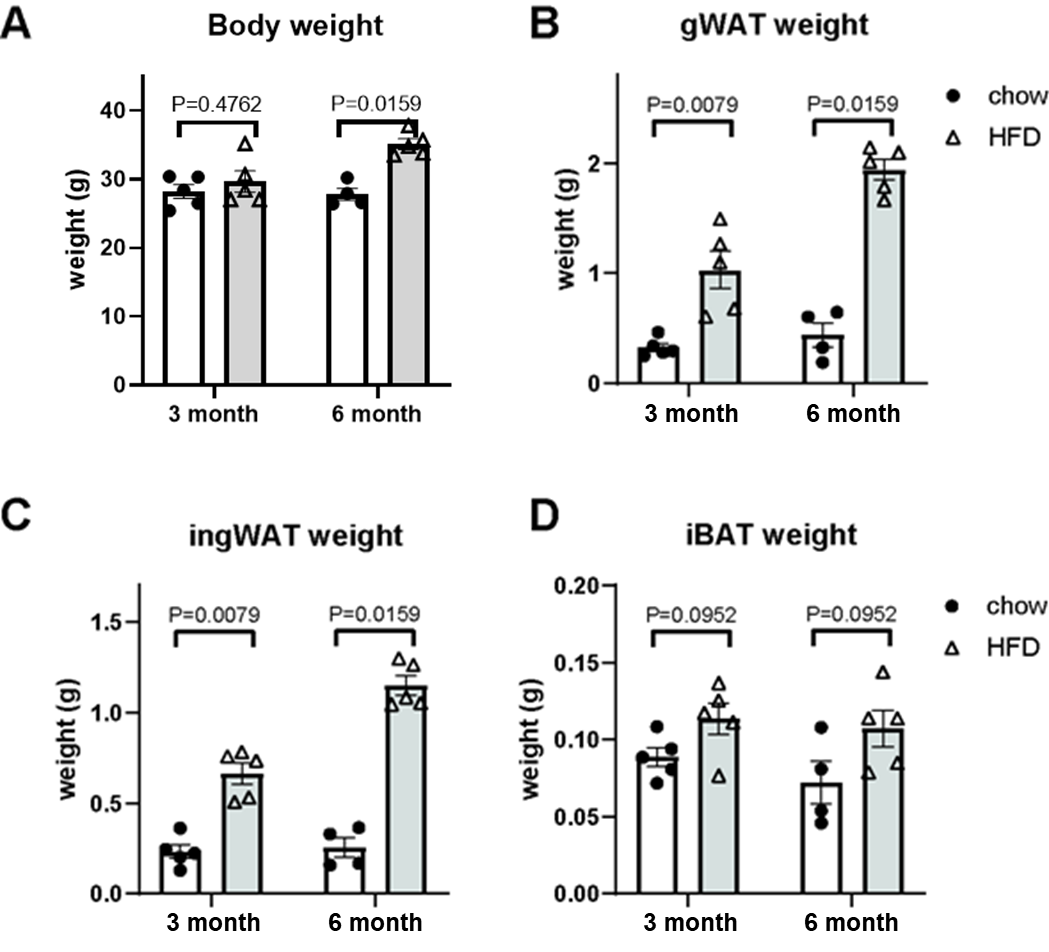


Figure S1. Body weight and the weights of gonadal white adipose tissue (gWAT), inguinal white adipose tissue (ingWAT), interscapular brown adipose tissue (iBAT) from mice fed with chow and high fat diet (HFD). A. Body weight of mice after 3 month or 6 months feeding with chow and high fat diet. B-D. The weight of gWAT (B), ingWAT (C), iBAT (D) of mice after 3 month or 6 months feeding with chow and high fat diet. For data in all panel: chow 3 month: n = 5, HFD 3 month: n = 5, chow 6 month: n = 4, HFD 6 month: n = 5.


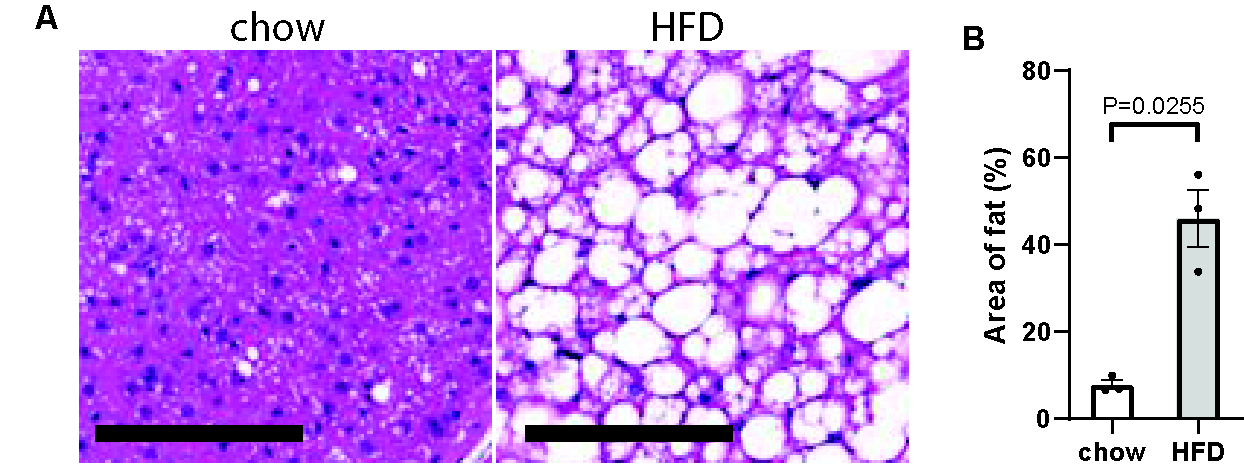


Figure S2. Whitening of brown adipose tissue in high fat diet (HFD) fed mice. A. HE staining of brown adipose tissue from mice fed with chow and HFD. Scale bar: 100 μm. B. Quantification of fat area coverage in HE staining images. For each group: n = 3.


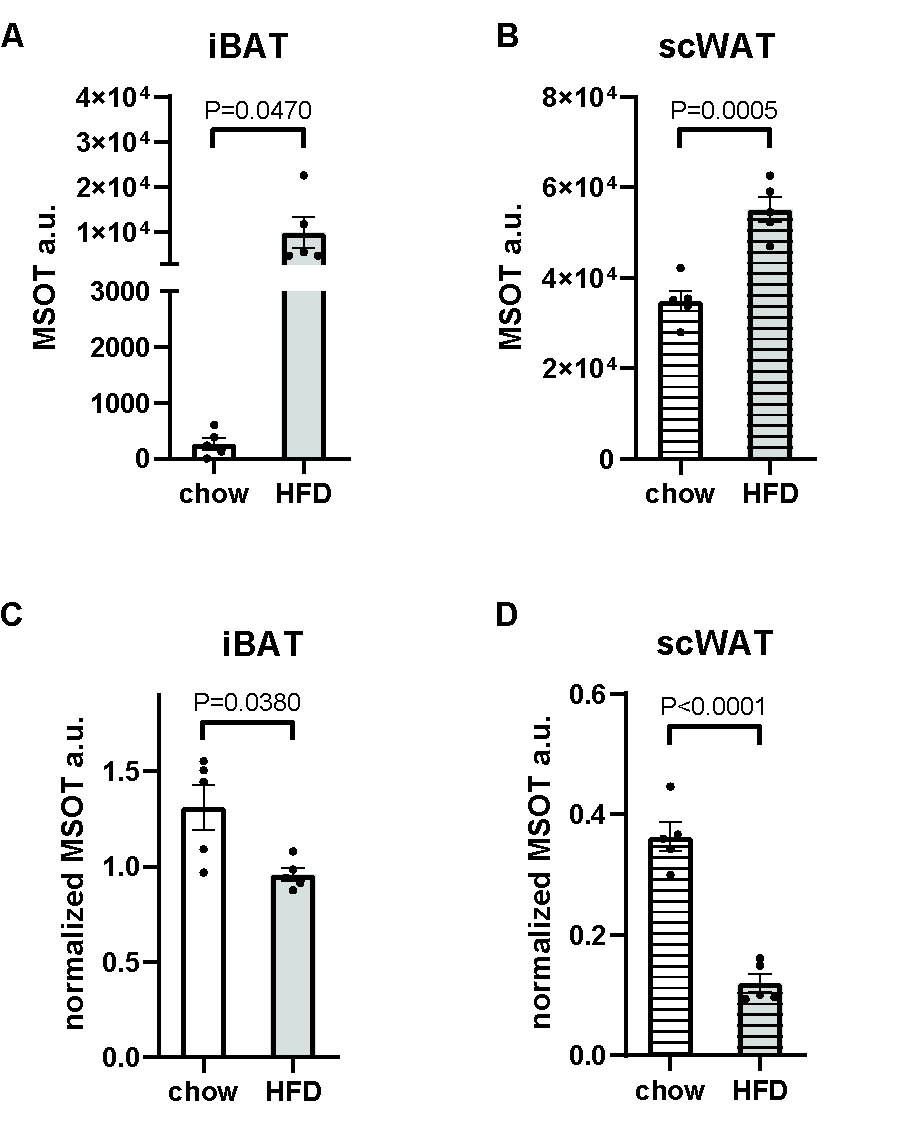


Figure S3. MSOT unmixing results of lipid and total blood volume (TBV) of interscaplular brown adipose tissue (iBAT) and subcutaneous white adipose tissue (scWAT). A-B. Lipid unmixing results from iBAT (A) and scWAT (B). C-D. TBV unmixing results from iBAT (C) and scWAT (D). For all groups in each panel: n = 5.

Figure S4. A. Body weight of mice fed with chow or high fat diet (HFD) with or without exercise. Chow sedentary: n = 5, chow exercised: n = 6, HFD sedentary: n = 4, HFD exercised: n = 4. B. dWAT thickness of female mice group measured by RSOM. Chow sedentary: n = 5, chow exercised: n = 5, HFD sedentary: n = 5, HFD exercised: n = 5.


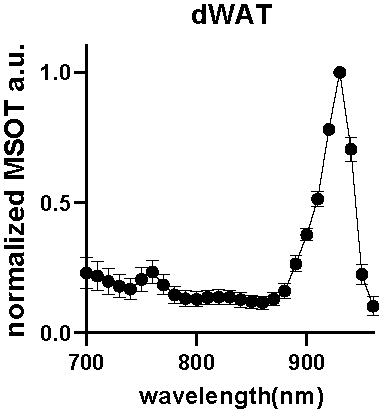


Figure S5. Normalized spectra of dermal white adipose tissue (dWAT) from normal mice. n = 5.


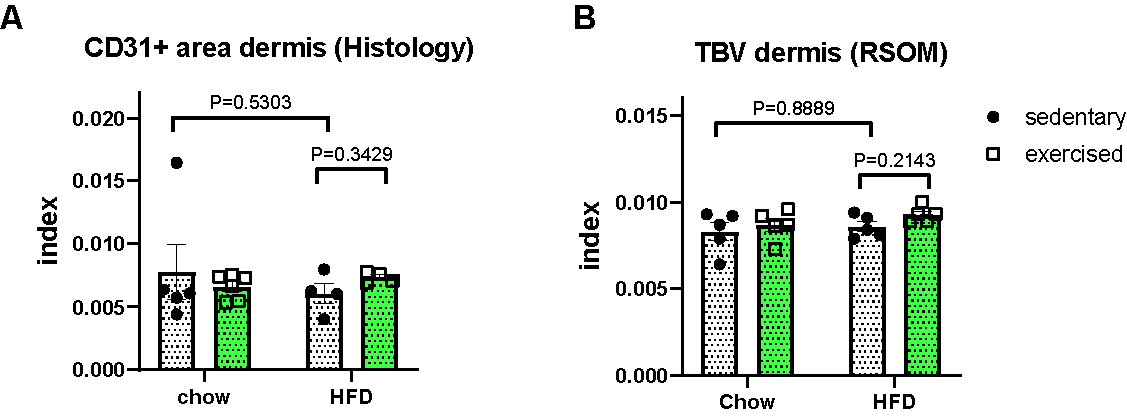


Figure S6. Vessel density in dermis of mice fed with chow or high fat diet (HFD) with or without exercise. A. CD31+ area coverage in dermis of mice fed with chow or high fat diet (HFD) with or without exercise. Chow sedentary: n = 5, chow exercised: n = 6, HFD sedentary: n = 4, HFD exercised: n = 4. B. RSOM result of total blood volume (TBV) in dermis of mice fed with chow or high fat diet (HFD) with or without exercise. For each group: n = 5


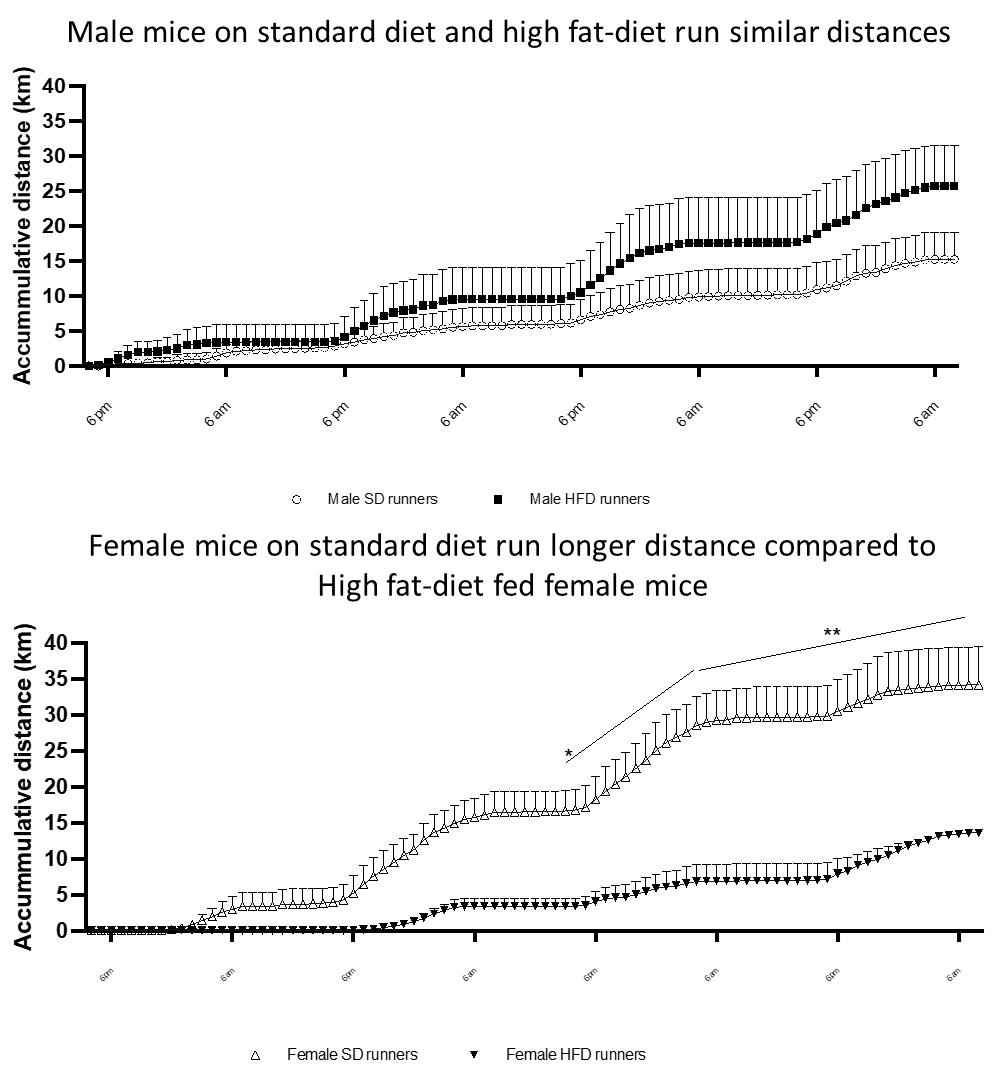


Figure S7. The running wheel data of the male and female in the standard diet and high fat-diet mice groups.
